# A mathematical function for the description of nutrient-response curve

**DOI:** 10.1371/journal.pone.0187292

**Published:** 2017-11-21

**Authors:** Hamed Ahmadi

**Affiliations:** Bioscience and Agriculture Modeling Research Unit, Department of Poultry Science, Tarbiat Modares University, Tehran, Iran; Universidad de las Palmas de Gran Canaria, SPAIN

## Abstract

Several mathematical equations have been proposed to modeling nutrient-response curve for animal and human justified on the goodness of fit and/or on the biological mechanism. In this paper, a functional form of a generalized quantitative model based on Rayleigh distribution principle for description of nutrient-response phenomena is derived. The three parameters governing the curve a) has biological interpretation, b) may be used to calculate reliable estimates of nutrient response relationships, and c) provide the basis for deriving relationships between nutrient and physiological responses. The new function was successfully applied to fit the nutritional data obtained from 6 experiments including a wide range of nutrients and responses. An evaluation and comparison were also done based simulated data sets to check the suitability of new model and four-parameter logistic model for describing nutrient responses. This study indicates the usefulness and wide applicability of the new introduced, simple and flexible model when applied as a quantitative approach to characterizing nutrient-response curve. This new mathematical way to describe nutritional-response data, with some useful biological interpretations, has potential to be used as an alternative approach in modeling nutritional responses curve to estimate nutrient efficiency and requirements.

## Introduction

A systematic relationship between the dietary concentrations or intake levels of a nutrient and the degree of the response is often observed. Description of the nutrient-response relationship requires a model which takes these observations into account [1]. The models should be able to describe different types of nutrients and nutrient sources, wide ranges of nutrient level, and the different types of animal and human responses. The calculated parameters of the model may be used to calculate the reliable estimates of nutrient-response traits, such as response at zero levels, biological efficiency, and requirement of nutrients.

Several biological, mathematical and statistical models involved fitting a non-linear logistic [2] and exponential [3] shape, enzyme kinetic [4, 5] with parabolic shape, and broken line [3] have been proposed to describe nutrient-response trajectories for animals. All these approaches were justified based on the goodness of fit and/or on the biological mechanism. The comparison has been performed with different models and at many levels of nutrients, starting from the ability of the model presented to fit nutritional data by performing different statistical tests and taking into account the complexity of the models considered. An example of this kind of work (derivation and comparison of several nutritional model) is [6] were linear, quadratic, broken line and saturating models are proposed and compared.

Lord Rayleigh [7] introduced the Rayleigh distribution in connection with a problem in the field of acoustics. Until recently, numerous works have been done related to this distribution in different areas of science. However, the application of Rayleigh distribution paradigm to describe the nutritional responses of animals has not been reported.

In this paper, a functional form of a generalized quantitative function based on the Rayleigh distribution principle for description of the nutrient-response phenomena is drive and expand. The derived function was then applied to the interpretation and description of several nutritional experiments in which different animal, chick and/or human were fed diets varying in concentration of nutrients. The parameters governing the curve provide the basis for deriving relationships between the nutrient and the physiological responses and explain the nutrients and responses with numerical indexes. In addition, an evaluation and comparison were performed based on experimental and simulated nutritional data sets to check the suitability of new derived and four-parameter logistic model for describing nutrient responses.

## Methods

### Function derivation

In probability theory and statistics, the Rayleigh distribution [7] is a continuous probability distribution for positive-valued random variables. The probability density function of the Rayleigh distribution for a random variable *x* is [8]:

$$f(x|g)\;=\;\frac{x}{\;g^{2}\;}e^{-(\frac{{\;x}^{2}}{{2\;g}^{2}\;})}\;for\;x\geq 0$$ (1)

where *g* is the continues scale parameter of the distribution (*g>0*). Integrating Eq 1 yields the cumulative distribution function of the form:

$$F(x|g)\;=\;1-{\;e}^{-(\frac{{\;x}^{2}}{{2\;g}^{2}\;})}$$ (2)

Output of this function increases from 0 to 1 when *x* increases from 0 to +∞. Modification of Eq 1 by defining an additional parameter, *m*, as the asymptotic response at *x→+∞*, gives:

$$F(x|g,m)\;=\;m-{m\;e}^{-(\frac{{\;x}^{2}}{{2\;g}^{2}\;})}$$ (3)

For x≥0 the range of function is from 0 to *m*. Since the Eq 3 requires that *Y = m-1* when, *x = 0* this equation may not be able to calculate intercept at suitable point required by the experimental data. Modification of the Eq 3 by translation of the ordinate axis, the new mathematical function then becomes:

$$F(x|g,m,b)\;=\;m+(b-m){\;e}^{-(\frac{{\;x}^{2}}{{2\;g}^{2}\;})}$$ (4)

where *b* is the ordinate intercept. These transformations increase the utility of the model since parameter *m* (maximum theoretical response when the level of a nutrient is not limiting) and *b* (response at zero nutrient level) are the points that represent two important measurable and interpretable values.

To the development of a most interpretable general equation for nutritional responses, two criteria should be considered [1, 5]: first, since the majority of nutritional responses appear to follow the sigmoidal kinetics, the general equation must be able to treat the nonlinear asymptotic sigmoidal behavior, and secondly, the theoretical curve obtained by the general equation must be able to cross the ordinate axis at any point based on the experimental data. Modification and transformation of the Rayleigh distribution yields the general model satisfying both of the proposed criteria.

In practice, it is convenient to make some alterations when applying Eq 4 to the results of a nutritional experiment. Thus, Eq 4 may be re-written as:

$$Y\;=\;R_{max}+(R_{min}-R_{max}){\;e}^{-(\frac{{\;x}^{2}}{{2\;E_{max}}^{2}\;})}$$ (5)

The parameters are defined as follows:

*Y* = experimental response to investigating nutrient

*x* = dietary nutrient concentration or intake,

*R*_*min*_ = intercept on response axis (response at zero dietary nutrient concentration or intake),

*R*_*max*_ = asymptotic maximum theoretical response (response at high nutrient concentration),

*E*_*max*_ = shaping parameter that locates the inflection point.

The point where the rate of the response changes from an increasing to a decreasing slope function is called the inflection point. In the present mathematical function the inflection point occurs at a nutrient level or intake of. *E*_*max*_. This parameter may also be used to determine the biological efficiency of a nutrient source, i.e., the relative ability of that source to produce a desired response. The response at the nutrient level of *E*_*max*_ (*RE*_*max*_) can then be calculated as:

$${RE}_{max}\;=\;0.606\;(R_{min}-R_{max})+R_{max}$$ (6)

Nutrient requirements may be stated for any predictable response. Setting Eq 5 equal to *0*.*95×R*_*max*_ and solving for *x*, it is possible to estimate the nutrient requirement (*Req*):

$$Req\;=\;E_{max}\;\sqrt{\text{log}\;\left(\frac{{\left(1-0.95\right)}^{2}{{\;R}_{max}}^{2}}{{\left(R_{max}-R_{min}\right)}^{2}}\right)}$$ (7)

The proportions of the asymptote, 95% is arbitrary value determined to be reasonable through experience in several studies designed for estimating the nutrient requirements [9, 10]. The value of 0.95 in Eq 7 may be replaced with 0.90 or 0.99 to estimate the nutrient requirement at 90 or 99% of maximum response, respectively.

The dietary nutrient concentration or intake required for maintenance (*x*) can be obtained by solving the Eq 5 when *Y = 0* with respect to *x*:

$$x_{m}\;=\;1.414\;\sqrt{{E_{max}}^{2}\;\text{log}\;\left(1-\frac{R_{min}}{R_{max}}\right)}\;for\;R_{min}\leq 0$$ (8)

### Mathematical model fitting

**Fig 1:**
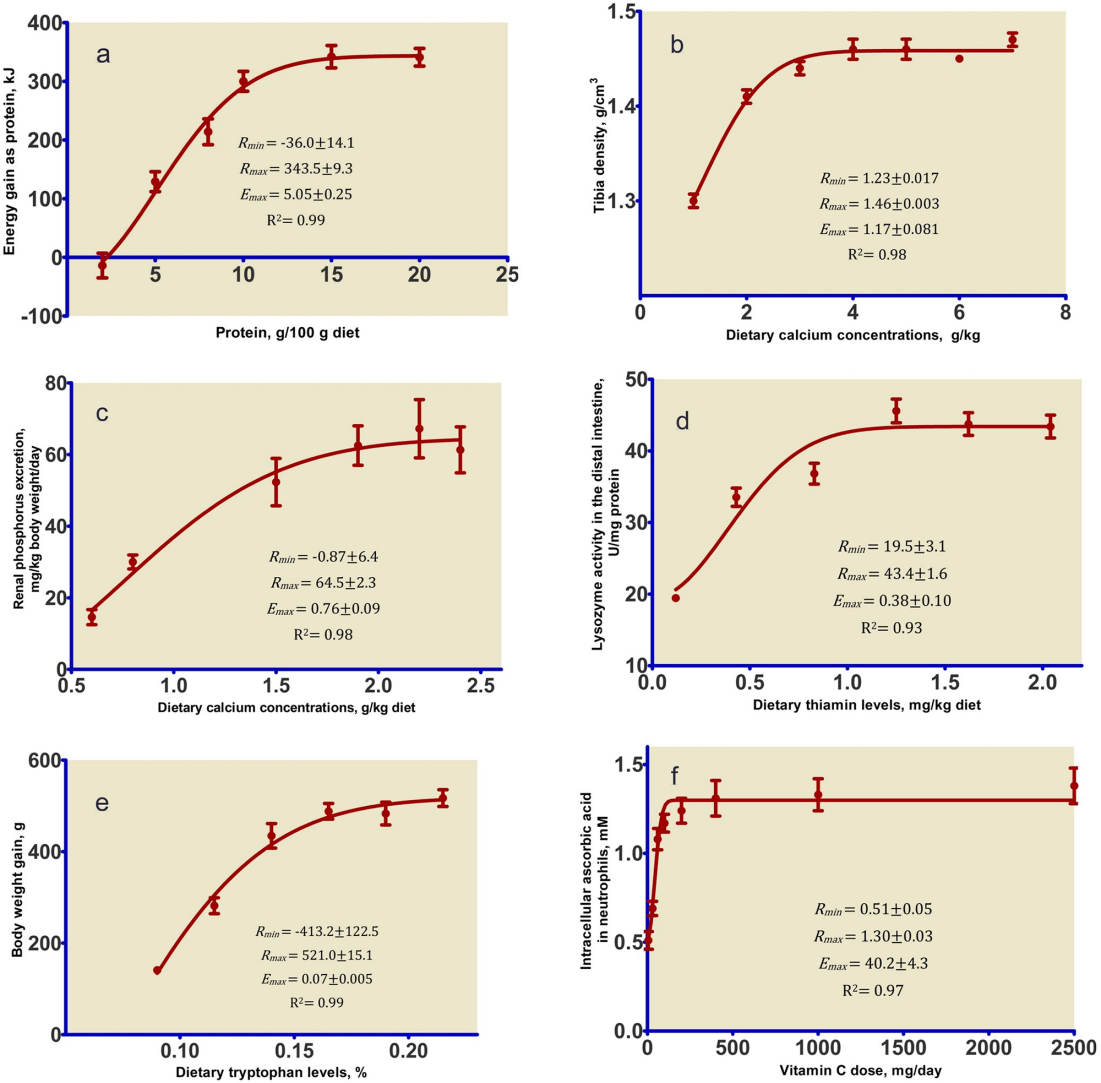
The theoretical nutrient-response curve calculated from Eq 5 fitted to experimental data. Values for model parameters (mean± standard error) defining the theoretical curve are shown in the inset. **a)** Energy balance of rats fed various levels of dietary protein for 2 weeks. Values are means ± standard error, n = 9. Data are taken from [11]. **b)** Tibia density of weanling rats fed incremental calcium concentrations for 13 weeks. Values are means ± standard error, n = 3. Data are taken from [12]. **c)** Renal phosphorus excretion of cats fed a diet with different levels of di-calcium phosphate. Values are means ± standard error, n = 10. Data are taken from [14]. **d)** Lysozyme activity in the distal intestine of young grass carp fed graded levels of thiamin. Values are means ± standard error, n = 6. Data are taken from [16]. **e)** Body weight gain of chicks fed diets containing graded levels of tryptophan at moderate (25°C) temperatures from 7 to 21 d of age. Values are means ± standard error, n = 9. Data are taken from [15]. **f)** Intracellular ascorbic acid concentrations in neutrophils as a function of vitamin C dose for healthy young women. Values are means ± standard error, n = 13 at doses 0–200 mg daily, n = 11 at doses 400 and 1,000 mg daily, and n = 10 at 2,500 mg daily. Data are taken from [13].

Testing the applicability of the derived mathematical model was done in two steps on the basis of the published data obtained from 6 studies [11–16] and simulated data sets generated by the software. At the first step, the function (Eq 5) was used to analyze the data of the nutritional experiments for 5 animal, chick and human, 5 nutrients (protein, calcium, thiamin, tryptophan, and vitamin C), and 6 responses (energy balance, bone quality, nutrient excretion, enzyme activity, weight gain, and intracellular nutrient concentration), summarized in Table 1 and illustrated in Fig 1. At the second step, the simulated data were generated using a procedure namely *randn* which is a standard framework of Random Number Generation provided by Matlab software (Matlab 2015a, MathWorks, Natick, MA). A total of 10 data sets were created for a nutritional scenario. Parameters for the simulated data were obtained from an experiment conducted to study the response of laying hens (Hy-Line W36) during initial phase of egg production (31 to 38 weeks of age) subjected to different intakes of dietary threonine. The main purpose of this experiment [conducted in our institute by H. Ahmadi and his collogues, data are presented in the Supporting Information (See S1 Table; sheet entitled “Hens Original Data”)] was to determine the requirements for threonine of laying hens. Seven levels of dietary threonine ranging from 3.2 to 5.6 g/kg diet (3.2, 3.6, 4.0, 4.4, 4.8, 5.2, and 5.6 g/kg diet) were fed in the experiment. Egg mass (g/bird/day) of hens from 31 to 38 weeks was considered as the dependent variable and dietary threonine intake (mg/day) was considered as the independent variable. The egg mass was calculated by multiplying percentage egg production by egg weight for each replicate. The average egg mass and dietary threonine intake of each pen (groups of 8 birds) within diet and replicate was drawn from a normally distributed pseudorandom numbers with a mean of and a standard deviation obtained from real hen’s response. Fifty replicates (pens) were simulated for each diet. The simulation process was replicated 10 times to generate 10 separate data sets for response of laying hens to dietary threonine. An excel spreadsheet including the generated data sets may be found in Supporting information (See S1 Table; sheet entitled “Simulated data & fitting values”). The two models were fitted to the all 6 experimental and 10 simulated data sets to assess whether the new derived model (Eq 5) makes other commonly used model i.e. the four-parameter logistic model superfluous. Four-parameter logistic equation is one of the most commonly used functions to describe the response to increasing dietary nutrient concentration. The form of equation as described by [2] was used:

$$Y\;=\;\frac{R_{max}+[b(1+c)-R_{max}]{\;e}^{-kx}}{x+c{\;e}^{-kx}\;}$$ (9)

The parameters are defined as follows: The *Y* variable (dependent variable) is the response to the nutrient. The variable *x* (predictor variable) is the dietary nutrient concentration or intake, *R*_*max*_ is asymptotic maximum theoretical response, *b* is intercept on *Y* axis, *c* is shaping parameter that locates the inflection point, and *k* is scaling parameter that scales *x*. Experimental and simulated nutrient-response data were fitted to Eq 5 using the standard method of the nonlinear curve-fitting techniques using PROC NLMIXED of SAS software (SAS software for Windows version 9.4, SAS Institute Inc, Cary, NC). The derived parameters (*R*_*min*_, *R*_*max*_, *E*_*max*_)) were used to generate a theoretical response curve and to calculate other indexes of nutrition characterization. Goodness of fit statistics were calculated to assess the model fits using two criteria; 1: proportion of variation accounted for (R^2^) which was calculated as *1-MSE/S*^*2*^_*y*_, where *MSE* is the residual mean square and *S*^*2*^_*y*_ is the total variance of the Y-variable, and 2: the RMSE values which is the root of MSE. The ranges are 0 ≤ R^2^ ≤ 1 and 0 ≤ RMSE ≤ +∞. Values closer to 1 and 0 indicate better fit in term of R^2^ and RMSE respectively. Under normality assumptions, the performance of models was also evaluated by the information criteria based on likelihood function which penalize the model with a greater number of free parameters. In this way, the Akaike’s information criteria (AIC), corrected version of AIC (AICc), and Schwarz or Bayesian information criterion (BIC) were calculated as follow [17]:

AIC=2f(ϕ)+2p

AICc=2f(ϕ)+2pn/(n-p-1)

**Fig 2:**
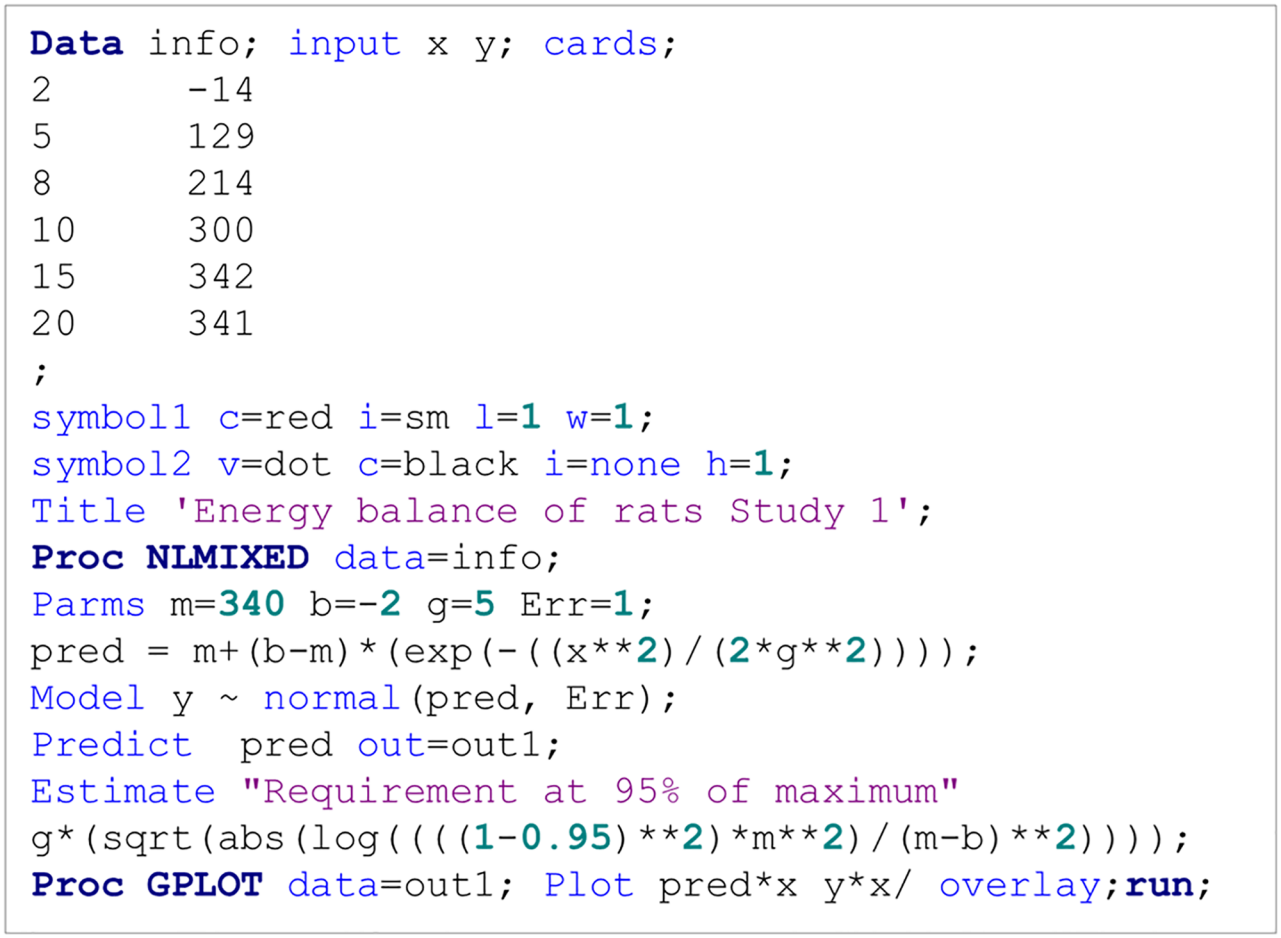
Example data and SAS (SAS software for Windows version 9.4, SAS Institute Inc, Cary, NC) statements used to fit the three-parameter function.

BIC=2f(ϕ)+plog(s)

where *f* is the negative of the marginal log-likelihood function, *ɸ* is the vector of parameter estimates, *p* is the number of parameters, *n* is the number of observations, and *s* is the number of subjects (SAS Institute Inc., 2009). Small values of these statistics indicate a better fit of the model to the data among two models. As an example, a sample of SAS program statements used to fit the derived function on data from study 1 is depicted in Fig 2. These statements can be used to apply the investigated model and analyze nutritional data.

**Table 1:** Data sources used in the study.

Study number	Nutrient	Range of nutrient	Response	Range of response	Animal or human	Figure	Reference
**1**	Protein	2−20 g/100 g diet	Energy gain as protein	-14−342 kJ	Rat	1a	[11]
**2**	Calcium	1−7 g/kg diet	Tibia density	1.3−1.47 g/cm^3^	Rat	1b	[12]
**3**	Calcium	0.6–2.4 g/kg diet	Renal phosphorus excretion	14.6−61.3 mg/kg body weight/day	Cat	1c	[14]
**4**	Thiamin	0.12−2.04 mg/kg diet	Lysozyme activity in the distal intestine	19.4−43.4 U/mg protein	Fish	1d	[16]
**5**	Tryptophan	0.09−0.215% of diet	Body weight gain	140.9−517.1 g	Chick	1e	[15]
**6**	Vitamin C	5−2500 mg/day	Intracellular ascorbic acid in neutrophils	0.51−1.38 mM	Human	1f	[13]

## Results

The experimental data and respective response curve (calculated using the Eq 5) for the 6 experiments are shown in Fig 1. All curves exhibit the threshold, increase and plateau of a typical nutrient-response relationship. The parameter estimates determined by this new fitting and application of the mathematical description to dose-response data, defining the theoretical curve, are shown in the inset of Fig 1. An acceptable (in term of R^2^) fit was obtained when using the model (Fig 1). The nutrient doses required to reach 90, 95, and 99% of maximum response (respective *R*_*max*_) were estimated according to Eq 7. When calculated *R*_*min*_*<0*, the dietary nutrient doses required for maintenance were obtained using Eq 8. Results of requirements and *x*_*m*_ are shown in the caption of Fig 1a to 1f. In study 1, a bioassay trial was done to evaluate energy balance of rats fed various levels of dietary protein for 2 weeks [11]. Protein requirements to reach 90, 95, and 99% of maximum energy gain as protein were estimated at 11.1±0.5, 12.6±0.6, and 15.5±0.7 g/100 g diet, respectively. Protein required for maintenance (*x*_m_) was 2.25±0.37 g/100 g diet (Fig 1a). In study 2, tibia density of weanling rats fed incremental calcium concentrations for 13 weeks was determined [12]. Dietary calcium requirements of young chicks to reach 90, 95, and 99% of maximum body weight gain were estimated at 1.10±0.06, 1.76±0.09, and 2.74±0.16 g/kg, respectively (Fig 1b). In study 3, values for renal phosphorus excretion of cats fed diets with different levels of di-calcium phosphate were presented [14]. The 90, 95, and 99% of maximum renal phosphorus excretion were estimated at 1.64±0.16, 1.87±0.19, and 2.31±0.25 g/kg of dietary calcium, respectively. Calcium required for maintenance was 0.12±0.45 g/kg diet (Fig 1c). In study 4, a growth test was done to measure the lysozyme activity in the distal intestine of young grass carp fed graded levels of thiamin [16]. The 90, 95, and 99% of maximum lysozyme activity in the distal intestine of young grass carp were estimated at 0.71±0.17, 0.84±0.21, and 1.08±0.28 mg/kg of dietary thiamin, respectively (Fig 1d). In study 5, a bioassay experiment was done to determine body weight gain of chicks fed with diets containing graded levels of tryptophan at moderate (25°C) temperature from 7 to 21 d of age [15]. Dietary tryptophan requirements of young chicks to reach 90, 95, and 99% of maximum body weight gain were estimated at 0.16±0.007, 0.18±0.009, and 0.22±0.012%, respectively. Tryptophan required for maintenance was 0.073±0.004% of diet (Fig 1e). In study 6, intracellular ascorbic acid concentrations in neutrophils as a function of vitamin C dose for healthy young women were measured [13]. The recommended dietary allowance for young women to reach 90, 95, and 99% of maximum response were estimated at 76.4±7.7, 89.9±9.2, and 115.2±12.1 mg/day, respectively (Fig 1f). Values of calculated AIC, AICc, BIC, R^2^ and RMSE for new three-parameter and four-parameter logistic models when fitted to nutritional data are shown in Table 2. Generally, the statistics indicate that both models could fit the data well. In all cases where the data (both actual and simulated) were analyzed by new model, the BIC values suggested that the new model is preferable in compared to logistic model.

**Table 2:** Values of calculated Akaike’s information criteria (AIC), corrected version of AIC (AICc), and Schwarz or Bayesian information criterion (BIC), R^2^ and root mean square error (RMSE) for new three-parameter and four-parameter logistic models when fitted to experimental and simulated nutritional data.

Data sources (Study number taken from Table 1)	New three-parameter model					Four-parameter logistic				
	AIC	AICc	BIC	R^2^	RMSE	AIC	AICc	BIC	R^2^	RMSE
**1**	55	95	55	0.99	12.47	57	117	56	0.99	11.82
**2**	-42	-22	-42	0.98	0.01	-43	18	-43	0.99	0.01
**3**	37	77	36	0.98	2.70	38	98	37	0.98	2.41
**4**	35	75	35	0.93	2.36	34	94	33	0.96	1.75
**5**	59	99	58	0.99	16.08	55	115	54	0.99	10.04
**6**	17	-3	-16	0.97	0.05	-16	14	-15	0.97	0.05
**Simulated data set 1**	2121	2121	2136	0.86	4.95	2122	2122	2141	0.86	4.95
**Simulated data set 2**	2119	2119	2134	0.86	4.94	2120	2121	2140	0.86	4.94
**Simulated data set 3**	2118	2118	2134	0.86	4.94	2117	2117	2136	0.86	4.91
**Simulated data set 4**	2079	2079	2094	0.87	4.67	2078	2078	2097	0.87	4.65
**Simulated data set 5**	2120	2120	2136	0.85	4.95	2120	2120	2139	0.85	4.94
**Simulated data set 6**	2135	2135	2150	0.85	5.06	2134	2135	2154	0.85	5.04
**Simulated data set 7**	2066	2066	2082	0.87	4.58	2066	2066	2085	0.87	4.57
**Simulated data set 8**	2112	2112	2127	0.86	4.89	2112	2112	2132	0.86	4.88
**Simulated data set 9**	2135	2135	2150	0.85	5.06	2134	2134	2154	0.85	5.04
**Simulated data set 10**	2078	2078	2093	0.87	4.66	2077	2077	2096	0.87	4.64

## Discussion

Several non-linear curve-fitting algorithms are available and satisfactory for fitting the three-parameter model. In this work, a standard procedure of the nonlinear least squares was applied by means of using GAUSS method developed in SAS software (SAS software for Windows version 9.4, SAS Institute Inc, Cary, NC). The GAUSS method is one of the most commonly integration method used in biological research [18]. It was found, while working with the model and data, that this procedure gives a rapid convergence in fitting the model to the all 6 data sets when there are good starting estimates available for the three parameters.

A very large number of non-linear models have been fitted to nutritional response data. The two models compared here, the new three-parameter model, and a four-parameter logistic model. The calculated AIC, AICc, and BIC for the competing new three-parameter model were in most of cases lower than those for the four-parameter logistic model. So, if a nonlinear model with fewer free parameters fits the data (nearly) as well as another one with more parameters, the simpler model is to be preferred. The current analysis contains two group of data sets (first group includes 6 actual nutritional experiments, and second group includes 10 sets of real-based simulated data) where the new proposed two-parameter model has nearly equal R^2^ values indicating the new model performs as good as the logistic model. Considering the information criteria values, most data sets show a significantly better fit for the two-parameter model, so it is reasonable to count this model as an alternative tool to nutritional studies. Overall results in this study revealed that model selection criteria favoring three-parameter model over logistic ones.

An appropriate quantitative mathematical relationship produces numerical indexes of characterization which gives new insight into the utilization of a nutrient. Any mathematical-nutritional model must have biological significance in that it has a theoretical basis in biological structure and function [19]. Establishing a minimum response or response at zero dietary nutrient level or intake (*R*_*min*_), a maximum response or response at very high nutrient concentration (*R*_*max*_) and an inflection point or biological efficiency of a nutrient (*E*_*max*_) is useful to determine the nutrient response curve in three interpretable quantities. These parameters establish limits on the efficacy of a nutrient in promoting a desired response [4, 19].

Requirement is defined as the dietary nutrient level necessary to produce a desired response level [3]. Choice of desired response level is arbitrary [10]. It is generally suggested that the nutrient-response curve may better be modeled as a continuous mathematical function with no "breakpoint". The breakpoint method is based on a statistical [4] (rather than mathematical model) product of the responses selected to fit the intersecting two (or more) lines. The requirement was taken as the abscissa of the breakpoint in the curve [3]. The estimated requirements by the breakpoint method could usually be considered low, because there is little margin for safety [4]. It is reported that the nonlinear models more accurately represent biological responses than models that force responses to conform to straight lines [3]. Interpretation of nutritional responses as Rayleigh distributed phenomena has important implications for the estimation of nutritional requirements. The new mathematical function provides a flexible tool to estimate the requirement at any desired response level, i.e., a desirable location on the response curve (proportion of *R*_*max*_). The model may help one to make a rational choice concerning the desired response level and nutrients needed to obtain that response.

Another important aspect of the modeling nutrition curve is the development of bioassay approach providing relevant comparisons of the biological efficiency of various forms of the nutrient sources [20]. The discussed function (Eq 5) generates two applicable constants allowing comparison between alternate nutrient sources, i.e., *R*_*max*_ and *E*_*max*_. Consider a case in which it is found that different sources of a nutrient yield the same *R*_*max*_; at high dietary level of nutrients, these sources may be considered equal. If dietary level of the nutrients are limiting, the nutrient source showing the lowest *E*_*max*_ would be preferred. The *E*_*max*_ is the dietary level or intake of the nutrients needed to achieve a response equal to *0*.*606 (R*_*min*_*-R*_*max*_*)+R*_*max*_. It indicates exactly where the physiological response is most sensitive to changes in the nutrient level. This parameter provides a most meaningful direct numerical comparison because each nutrient source is represented at its maximum rate of promotion of response. The nutrient with the lower *E*_*max*_ has the better marginal efficiency of utilization.

The use of the concept of Rayleigh distribution produces an equation, the three-parameter mathematical curve for nutritional responses, which this equation describe many experimental observations found in the nutrition literature. It also provides a new application of a statistical description for nutritional process. The derived mathematical function presented in this paper is a deterministic (no stochastic component), static model based on the concept of Rayleigh distribution. It is a theoretical model, but it describes a wide range of empirical observations (physiological responses in a large range of situations).

The function showed some suitable characteristics while it was fitted to the data. The estimated parameters have biological interpretation and they may be used to calculate reliable estimates of the nutrient response relationships, such as maximum or average performance due to graded level of a nutrient. Data over a wider range of the nutrient levels may be included in the analysis. Theoretical nutrient-response curves generated by the data are capable of predicting experimental responses with acceptable accuracy. The discussed model is also characterized by a) simplicity with only three parameters, b) flexibility due to its structure, and c) the easiness of the fitting procedure. This study indicates the derivation, usefulness and wide applicability of the new three-parameter mathematical curve function to formulate the relationship between levels of dietary nutrients and physiological responses. This study is design to drive a simple three-parameter model and to judge the capability of a new derived versus logistic models for nutrient response data sets. The presented results on model fits may help one to choose accurately among new and logistic models for future nutritional data. Furthermore, to facilitate the application of the model and model selection criteria, a functional SAS code implementation, which relies on SAS software is included to the paper. This new mathematical way to describe nutritional-response data, with some useful biological interoperations, has potential to be used as an alternative approach in modeling nutritional responses curve to estimate the nutrient efficiency and requirements.

## Supporting information

S1 Table Microsoft excel database containing all the simulated (10 sets) and experimental data used in this study. Excel sheets in order: The sheet entitled “Hens Original Data” contains the results of an experiment conducted to study the response of laying hens during initial phase of egg production subjected to different intakes of dietary threonine. The sheet entitled “Simulated data & fitting values” contains the 10 simulated data sets that were generated using a standard procedure of random number generator. The predicted values obtained by the new three-parameter and conventional four-parameter logistic models were also appeared in this sheet.
(XLSX)
